# Circulating tumor cells mirror bone metastatic phenotype in prostate cancer

**DOI:** 10.18632/oncotarget.25634

**Published:** 2018-06-29

**Authors:** Andreas Josefsson, Karin Larsson, Marianne Månsson, Jens Björkman, Eva Rohlova, Daniel Åhs, Helena Brisby, Jan-Erik Damber, Karin Welén

**Affiliations:** ^1^ Sahlgrenska Cancer Center, Department of Urology, Institute of Clinical Sciences, Sahlgrenska Academy, University of Gothenburg, Gothenburg, Sweden; ^2^ Department of Orthopaedics, Institute of Clinical Sciences, Sahlgrenska Academy, University of Gothenburg, Gothenburg, Sweden; ^3^ TATAA Biocenter AB, Gothenburg, Sweden; ^4^ Department of Anthropology and Human Genetics, Faculty of Science, Charles University, Prague, Czech Republic; ^5^ Laboratory of Gene Expression, Institute of Biotechnology CAS, BIOCEV, Vestec, Czech Republic; ^6^ Centre for Experimental Medicine, Institute for Clinical and Experimental Medicine, Prague, Czech Republic

**Keywords:** liquid biopsies, circulating tumor cells, skeletal metastases of prostate cancer

## Abstract

Circulating tumor cells (CTCs) are promising biomarkers in prostate cancer (PC) because they derive from primary tumor and metastatic tissues. In this study, we used quantitative real-time PCR (qPCR) to compare the expression profiles of 41 PC-related genes between paired CTC and spinal column metastasis samples from 22 PC patients that underwent surgery for spinal cord compression. We observed good concordance between the gene expression profiles in the CTC and metastasis samples in most of the PC patients. Expression of nine genes (*AGR2*, *AKR1C3*, *AR*, *CDH1*, *FOLH1*, *HER2*, *KRT19*, *MDK*, and *SPINK1*) showed a significant correlation between the CTC and metastasis samples. Hierarchical clustering analysis showed a similar grouping of PC patients based on the expression of these nine genes in both CTC and metastasis samples. Our findings demonstrate that CTCs mirror gene expression patterns in tissue metastasis samples from PC patients. Although low detection frequency of certain genes is a limitation in CTCs, our results indicate the potential for CTC phenotyping as a tool to improve individualized therapy in metastatic prostate cancer.

## INTRODUCTION

Prostate cancer (PC) is the most commonly diagnosed cancer and the sixth leading cause of cancer-related death among men worldwide. Bone metastasis is the leading cause of morbidity and mortality in patients with PC and is the preferred target for therapy. Because metastatic biopsies are often difficult to obtain, using blood samples would be preferred for biological characterization of PC. Classical blood biomarkers are used for prognostic evaluation of disease progression and therapeutic response. but analysis of circulating tumor cells (CTCs), cell-free DNA, cell-free RNAs or miRNAs, exosomes and thrombocytes in liquid biopsies can provide broader molecular details of the cancer phenotype, which could be used for personalized therapy with specific targeting drugs [1].

CTCs can be used as tumor biomarkers because a high number of CTCs strongly correlate with metastatic disease and poor prognosis in metastatic PC in both the hormone naïve (HN) and castration-resistant (CR) settings [2, 3]. Furthermore, detection of specific gene expression in CTCs can provide reliable information regarding the prognosis and therapeutic resistance to treatments targeting androgen receptor signaling [3–6]. CTCs may thus reflect not only the tumor burden but also the biology of the disease.

In various cancers, similarities have been detected in the genomic compositions of CTCs and tumor tissues [7, 8]. In PC, for example, shared genomic alterations have been identified in CTCs and tumor tissue samples [9]. Moreover, androgen receptor (AR) amplification is concordant between CTCs and tumor tissue biopsies from CRPC patients [10]. It is unclear, however, whether CTCs accurately reflect the phenotype of metastatic tissue in PC. Cho *et al.* showed that expression of a small number of genes (present or absent) was concordant between single CTCs and micro-dissected PC cells from bone marrow biopsies in 75% of cases [11]. In colorectal cancer, gene expression in the CTCs was more similar to that in liver metastases than the primary tumors, but the concordance between the gene expression profiles of CTCs and metastases was low [12]. In breast cancer, concordance of gene expression profiles in CTCs and primary tumors was highly variable [13].

In the present study, we analyzed the gene expression profiles in paired samples of CTCs and metastatic tissue from the spines from PC patients (Table 1). Our data show that to a large extent the gene expression profile of CTCs mirrors that in the paired metastatic tissue. However, all selected genes did not perform equally well. Therefore, careful selection of genes and analysis of a biomarker panel are required to develop CTC-based liquid biopsy strategies for clinical applications in PC.

**Table 1:** Patient characteristics

	At diagnosis		At surgery		Time from diagnosis to surgery (years)
Median age year (range)					
hormone naive (n=5)	68 (64-83)		68 (64-83)		0
GnRH naive (n=1)	82		87		5.8
GnRH initated (n=1)	69		70		0.1
CRPC (n=15)	70 (57-83)		75 (59-88)		4.5 (1.0-14.8)
Median PSA ng/ml (range)					
hormone naive (n=5)	731 (111-1200)		731 (111-1200)		
GnRH naive (n=1)	3.12		93		
GnRH initated (n=1)	1200		x		
CRPC (n=15)	33.5 (5-334)		81.4 (5.8-276)		
Gleason score (n)	GS 6-7		GS 8-10		GS X
hormone naive (n=5)	0		1		4
GnRH naive (n=1)	1		0		0
GnRH initated (n=1)	0		1		0
CRPC (n=15)^*^	7		6		2
Therapy before surgery	None	TAB^**^	TAB+Docetaxel	Docetaxel	Enzalutamide
CRPC (n=15)^***^	6	3	2	3	1

^*^ Gleason score was obtained at diagnosis, not at surgery.

^*^ TAB = total androgen blockade, GnRH analogue plus bicalutamide.

^**^ All patients were treated with GnRH analogues.

## RESULTS

### Detection of gene expression in spinal metastasis tissues and CTCs from PC patients

We excluded 15 of the 46 genes in the PC-panel from further analysis. These included *EPCAM* used for normalizing gene expression values, the general endogenous control genes *GAPDH* and *GUSB,* as well as *CD45* and *CD44* which mainly reflect the white blood cell contamination in the CTC samples. *MYC*, *TP53,* and *ANXA2R* were excluded due to their frequent detection in CTC-negative (*EPCAM*- negative) samples, which was interpreted as contaminating signals from the white blood cells (data not shown). Further, *ESR1*, *ESR2*, *PTCH1*, *MET*, *CYP11A1*, *CYP17A1*, and *CYP19A1* genes were excluded because they were rarely detected in the CTC samples (Table 2).

**Table 2:** Prostate cancer panel; detected gene expression signals in CTC and metastatic tissue and their correlation

Gene	Detected signals in CTC	Detected signals in metastases	Correlation coefficient, R	P-value		Detected signals in CTC	Detected signals in metastases
**AGR2**	18	22	**0.778**	**p<0.001**	**Genes not included in analysis due to low detection frequency in CTC samples**		
AHR	3	22	-0.500	0.667			
**AKR1C3**	17	22	**0.704**	**p<0.01**	CYP11A1	1	22
AKT2	11	22	-0.159	0.640	CYP17A1	1	14
ALDH	13	22	0.316	0.293	CYP19A1	1	22
**AR**	14	22	**0.565**	**p<0.05**	ESR1	0	22
**ARV7**	7	22	**0.750**	**0.052**	ESR2	0	13
AURKA	12	22	-0.130	0.688	MET	1	22
BCL2	10	22	-0.395	0.258	PTCH1	0	22
**CDH1**	14	22	**0.575**	**p<0.05**	**Genes not included in analysis due to frequent detection in CTC negative samples**		
CDH2	2	22	-	-			
DDR1	2	22	-	-	MYC		22
EGFR	4	22	-0.200	0.800	TP53		22
EMP2	11	22	0.359	0.278	ANXA2R		22
**FOLH1**	16	22	**0.900**	**p<0.001**	**Control genes not included in analysis**		
**HER2**	11	22	**0.712**	**p<0.05**	GAPDH	22	22
**KLK3**	18	22	**0.451**	**0.061**	GUSB	22	22
**KRT19**	16	22	**0.693**	**p<0.01**	CD44	22	22
**MDK**	18	22	**0.720**	**p<0.01**	CD45	2	2 22
**POU5F1**	13	21	**0.478**	**0.099**			
**PSCA**	10	22	**0.588**	**0.074**			
**RUNX2**	5	22	**0.872**	**0.054**			
SNAI1	5	22	0.300	0.624			
**SPINK1**	11	22	**0.724**	**p<0.05**			
SRD5A1	15	21	0.445	0.110			
TACSTD2	16	22	0.379	0.147			
TOP2A	14	21	0.263	0.363			
TUBB3	6	22	-0.029	0.957			
Twist1	7	22	0.179	0.702			
UPA	7	22	-0.414	0.355			
VEGFA	14	22	0.066	0.822			

Analysis are performed with Spearman rank correlation. Genes with a correlation between CTC and metastases with a p-value less than 0.1 are marked in bold text.

In the bone metastasis tissue, the 31 genes that were analyzed were detected in all samples, except for *POU5F1*, *SRD5A1,* and *TOP2A* (96%), *CYP17A1* (67 %), and *ESR2* (58 %). In the CTCs, the detection ratio of these 31 genes was much lower. The detection frequency in the CTC samples ranged from 87 % (*AGR2*, *AKR1C3*, and *KLK3*) to 4 % (*CYP11A1*, *CDH11*, *CYP17A1*, and *MET*) (Table 2). Prostate cancer origin of one of the CRPC patients could not be confirmed because the CTCs did not show any detectable expression of either KLK3 or FOLH1. Hence, this sample was excluded from further analyses.

### Identification of genes in CTC reflecting expression in bone metastases

We assessed the potential of the included genes in the CTC-based analysis to reflect gene expression in tissue metastases by estimating the correlation in signal intensity of the expression of individual genes in the CTC and metastasis samples of all patients. Nine of the thirty-one included genes showed a statistically significant correlation between the expression in CTC and the metastasis samples (Table 2). In addition, *ARV7*, *POU5F1*, *PSCA*, *RUNX2*, and *KLK3* showed moderate to a high degree of correlation (R>0.4 and p<0.100).

### Gene expression profiles in the CTCs and metastatic tissue samples of individual patients

We compared the gene expression profiles between matched CTC and metastatic tissue samples of each patient to get a broader picture of the metastasis than obtaining just the expression levels of individual genes. In this analysis, we included the calculated values for low detection levels in CTCs (i.e., cases with no signal for a specific gene despite sufficient CTC content). We analyzed 14 patients whose CTC samples showed gene expression values for more than 50% of the 31 included genes. Among these, 11 patients showed good correlation (correlation coefficient ≥ 0.5) between the gene expression profiles in CTC and metastasis samples. Six patients showed bootstrap generated p-values below 0.05 while five patients showed p-values between 0.05 and 0.1 (Table 3). Three of the 14 patients showed poor correlation (0.185, 0.250 and 0.307, respectively) between gene expression in CTC and metastatic tissue samples. Two of these three patients showed metastasis in the lungs (66%), whereas only one of the eleven patients (14 %) with a correlation coefficient > 0.5 showed lung metastasis.

**Table 3:** Correlation between gene expression in CTCs and metastases in individual patients

Patient ID	CTC/Mets pairs	Correlation coefficient, R	P-value
1	24	**0.532**	**<0.05**
2	29	**0.591**	**<0.05**
5	28	**0.508**	**<0.05**
6	28	**0.793**	**<0.001**
7	22	**0.676**	**<0.01**
9	18	**0.531**	**<0.05**
10	28	0.487	**<0.05**
12	20	**0.677**	**<0.01**
14	24	0.25	0.187
16	18	0.185	0.27
18	31	0.307	0.092
19	29	**0.593**	**<0.001**
21	30	**0.53**	**<0.001**
22	16	**0.506**	=0.05

Spearman correlations and bootstrap p-values for the gene expression profiles in CTCs and metastatic tissue in individual patients.

### Analysis of patient grouping based on CTC and metastatic gene expression profiles

**Figure 1:**
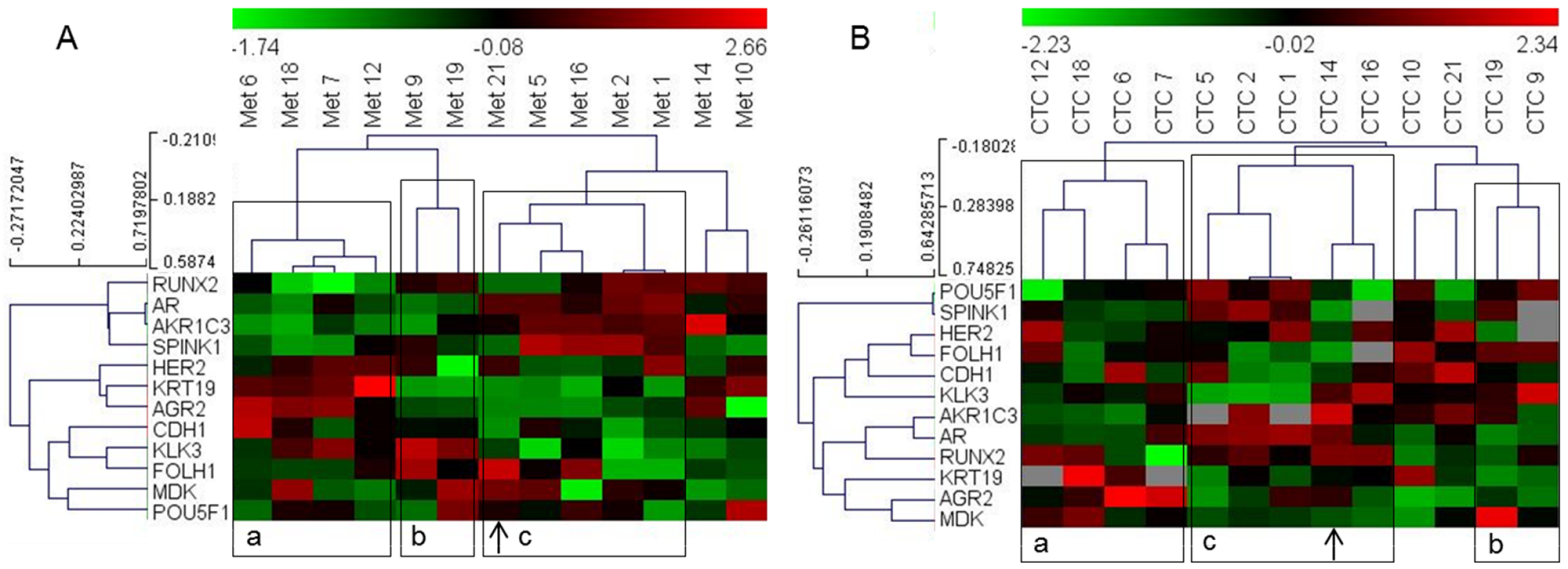
Heat map illustrates hierarchical clustering using significantly correlating genes in PC patients based on their expression in **(A)** metastatic tissue **(B)** and CTCs. Note: a, b and c represent groups of patient samples that cluster similarly in both metastases and CTC analyses. Arrows indicate samples switching clusters between the two analyses.

We selected the subset of 14 genes that showed good correlation between the CTC and metastatic tissue samples (Table 2) for hierarchical clustering analysis to determine whether patient grouping using the gene expression data from CTCs is similar to their grouping when the data from metastatic tissue samples were used. However, to optimize the clustering analysis, we only included patients with expression values for more than 10 of the included genes in their CTC samples, and we only included genes with positive expression signals in more than 10 of these patients. This resulted in a matrix of 13 patients and 12 genes (*ARV7* and *PSCA* genes were excluded). Based on expression in metastatic tissue samples, the patients were grouped into three clusters. Concurrent analysis using CTC-derived data showed that the eleven of the thirteen patients (exceptions: patient numbers 14 and 21) grouped similarly into three clusters (Figure 1).

**Figure 2:**
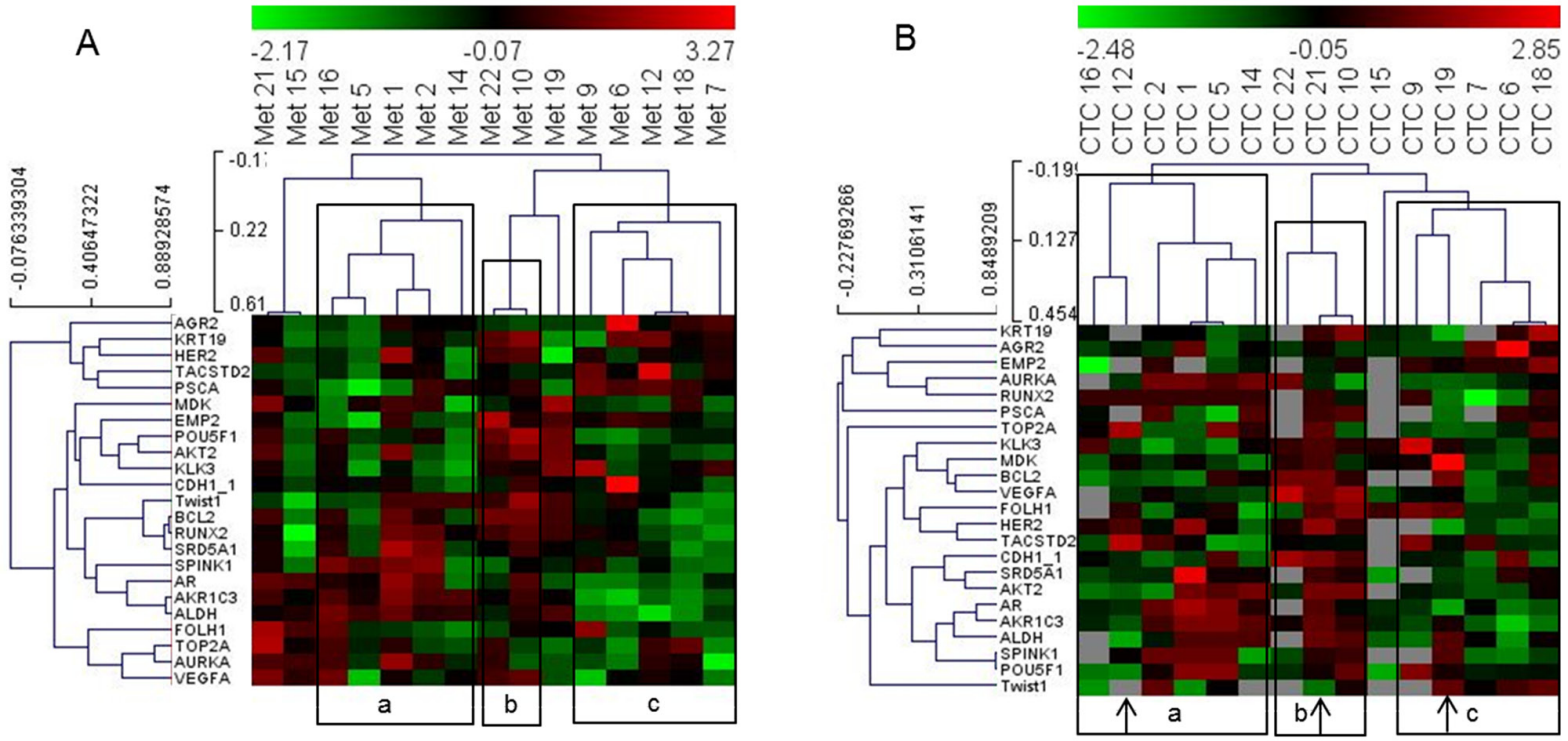
Heat map illustrates hierarchical clustering using significant genes without prior selection based on their expression in **(A)** metastatic tissue and **(B)** and CTCs. Note: a, b and c represent groups of patient samples that cluster similarly in analyses of both metastases and CTCs. Arrows indicate samples that switch clusters between the two analyses.

Next, we included all genes that were detected in more than 10 CTC samples and all patient samples whose CTC analyses resulted in 10 or more signals to determine the performance of the PC Panel in grouping patients based on CTC data without pre-analysis of significantly correlating genes or patients. We obtained a matrix of 15 patients and 23 genes. Hierarchical clustering of this dataset demonstrated that PC patient grouping based on the analyses of gene expression in CTCs was similar to that based on the metastatic dataset (Figure 2).

### CTC analysis reflects patient treatment status

**Figure 3:**
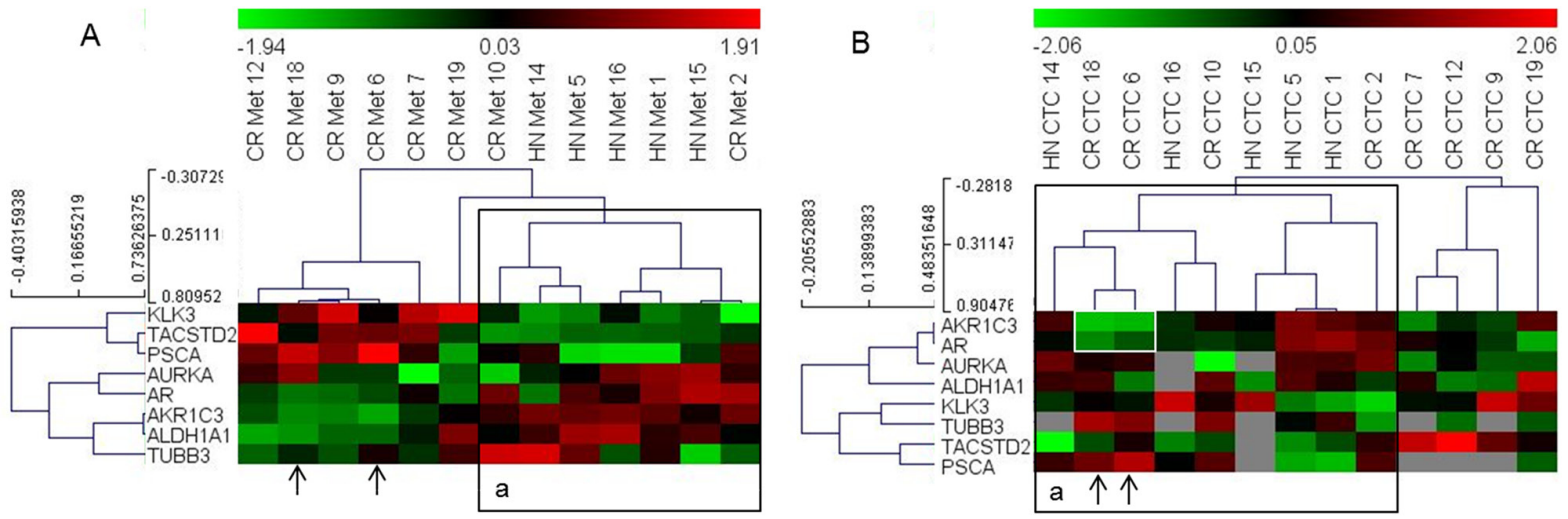
Heat map illustrates the hierarchical clustering using the limited gene subset discriminating HN and CR metastases, based on their expression in **(A)** metastases and **(B)** CTCs. Note: ‘A: a’ represents a cluster that includes all HN samples in the metastases analyses; ‘B: a’ represents a similar cluster in the CTCs. Arrows indicate samples that cluster differently in the CTC-based analysis; white box highlights the AR and AKR1C3 signals in two CR samples that change clusters.

Next, we analyzed if the gene expression data from the CTCs identified phenotypes that match the clinical status of the patients. To address this, we classified the patients based on their hormonal status at sampling, i.e., if they were resistant to treatment with GnRH analogues [castration-resistant (CR), n=12] or were hormone naïve (HN; hormone naïve and GnRH naïve, n=6, Table 1). For clustering analysis, we used nine genes that were differentially expressed in the metastasis samples of these two categories. However, due to the low detection frequency of certain genes in the CTC samples, the dataset was modified to include patients with genes that displayed more than 50% detection frequency in the CTC-derived data. Thus, we obtained a matrix of 8 genes and 13 patients (5 HN and 8 CR). Clustering of these patients based on the gene expression data from metastatic tissue samples revealed two clusters, of which one included all HN and two CR patients. Clustering analysis of the CTC-derived dataset from these patients again grouped all HN samples, with two more CR patients clustered together with the HN patients (Figure 3). Notably, the two CR patients that grouped with the HN cluster in the CTC-based analysis (18 and 6) displayed high expression (green signal) of both *AR* and *AKR1C3* genes, which was atypical for most HN samples (highlighted in Figure 3).

## DISCUSSION

CTCs have been used for a long time as biomarkers for PC prognosis and therapy response. However, the potential of obtaining phenotypical information from these circulating cancer cells has not been satisfactorily explored. In the present study, we analyzed the gene expression of CTCs to explore the potential of gaining phenotypical information regarding the metastatic disease.

The CTCs are intact cancer cells, and therefore carry biologically relevant information regarding the disease in addition to their use as biomarkers based on their enumeration. In the present study, we show a strong correlation between CTCs and bone metastasis samples from the same patient regarding the expression levels of several genes that are related to PC progression, metastasis, and therapy resistance. However, when analyzing the genes individually, it was obvious that the potential to reflect the expression level in metastases by CTC analysis differed among genes. Our analysis showed that nine out of forty-one genes displayed a strong correlation between their expression in CTCs and metastasis samples. Among these was *ARV7* which expression in CTCs is related to abiraterone and enzalutamide resistance in several studies [4–6]. Previously, detection of any of the other eight genes in CTCs has not been related to any clinically relevant characteristic, and is for the first time presented to be found in CTCs in prostate cancer patients. The *MDK* and *AGR2* genes are related to neuro-endocrine differentiation in CRPC [14, 15], which is a biological process that is associated with a non-functional AR signaling axis and correlates with poor prognosis. These tumors likely do not respond optimally to drugs that are targeted towards sustained AR signaling in CRPC. Similarly, although high expression of *AR*, *ARV7*, and *AKR1C3* indicates that the tumors are dependent on activated AR-signaling their overexpression may be a part of the activated resistance mechanisms that lower sensitivity to AR targeting drugs [5, 16]. This was illustrated by our findings that show higher expression of AR and AKR1C3 the CR patients than the HN patients.

There are at least two plausible reasons for the differences in expression of several genes between CTC and metastasis samples. First, these genes may be differentially regulated in a CTC than a metastatic tumor cell. Second, the CTC and metastasis samples may contain different amounts and types of contaminating cells. In the CTCs, leukocyte contamination affects the detection levels of certain genes, whereas, in the metastatic tissue, tumor stroma is a major contaminating factor that influences detection of gene expression from tumor cells alone. This is exemplified in the present study by the poor correlation of urokinase-type plasminogen activator (*UPA*) and vascular endothelial growth factor A (*VEGFA*) genes, which are both expressed by tumor cells as well as the tumor stromal cells. Thus, care must be taken in selecting genes that enable CTC analysis to provide useful information regarding the metastatic tumor cells.

In the individual patients, the gene expression profile of CTC samples correlated with the gene expression profile of the corresponding metastatic samples in most cases. However, we also encountered patients where the correlation was poor or absent. We are unable to reliably predict the reasons for these differences between patients because the patient material in this study was too small. However, the presence of lung metastasis in two out of three patients that showed poor concordance between gene expression in the CTCs and bone metastatic tissue samples indicates that these CTC samples may also represent lung metastases, which presumably represent a different phenotype than the bone metastases. This suggests that CTCs represent the whole metastatic disease, and therefore, the gene panel should potentially represent the biological characteristics of metastases in different organs. This is supported by previous findings that the single cell CTC mutational status may be highly heterogeneous [17].

Our study also showed that patient grouping based on gene expression analysis of CTC samples was similar to grouping based on the analysis of the metastatic tissue. Moreover, patients were grouped according to the hormonal status based on the gene expression profiles of both CTC and metastasis samples. This shows that although all independent genes may not display a good correlation between the CTC and metastasis samples, a combination of genes may represent clinically relevant information regarding the tumor phenotype.

Although the analysis of CTCs from liquid biopsies has many advantages, the technology requires further optimization to overcome the limitations. We encountered two technical issues in the present study. The first relates to the contamination by leukocytes during CTC isolation, which limits the expression panel to genes that are not expressed in leukocytes. This represents a major obstacle that needs improvement in CTC isolation methodologies to develop reliable treatment prediction analysis for immune-related therapies such as programmed death-ligand 1 (PD-L1) antibody-based immunotherapy. The second technical limitation was the reliance on EPCAM and HER2 antibodies for the isolation of CTCs. This limits the detection of CTC populations with low expression of these antigens [18] or those masking these antigens by macromolecules [19]. Hence, CTCs that were not isolated by our methodology may represent other sub-populations of CTC, and their genetic profile may also be critical for evaluating the status of PC metastasis. This problem may be overcome by novel isolation methods that are not based on epithelial antigens. Therefore, it is plausible that we may overcome the insufficient sensitivity to detect expression of certain genes in the CTC samples by utilizing other isolation methods.

De Bono *et al.* showed that the amount of CTCs reflects the progression and treatment response of PC [20]. Since one of the technical issues encountered with CTC profiling is sensitivity as a result of the limited amount of material, the usefulness of the method is dependent on the amount of CTCs that are available for detection. Thus, patients with a non-metastatic disease or those responding to therapy are less suitable for this type of analysis. The present study includes only patients with metastatic disease, but despite their severe condition, we observed significant variations in the amount of CTCs isolated from individual patients. This affected the detection frequency of certain genes depending on their relative expression levels and limited their use in the analyses.

Another challenge we encountered in our study was about interpreting the absent gene expression signals from samples with low CTC content. It was obvious from our results that some genes were less expressed than others despite comparable CTC content. Thus, an absent signal in CTC-derived expression data cannot simply be interpreted as a low expression signal because it may be a result of a too low CTC content to allow detection of the specific gene. In the present study, we developed a strategy to identify the threshold of CTC content that would enable detection of each gene individually and avoid false low detection values. If a gene was not detected in a sample despite sufficient CTC content, it was assigned an expression value lower than the lowest detected for that gene. If the CTC content was below the threshold and the gene expression could not be detected, that absent signal was excluded from further analysis. In the future, to enable expression profiling of CTCs for clinical applications, there is a need to increase the detection sensitivity and develop robust methods to handle absent gene expression signals as a result of limited CTC content.

In conclusion, our study demonstrates the potential of CTCs to mirror the gene expression profile of PC bone metastases in individual patients. The study also points out the importance of careful selection of genes to accommodate the technical and biological aspects that limit the interpretation of expression of different genes.

## MATERIALS AND METHODS

### Patients

We recruited twenty-five patients undergoing surgery for spinal cord compression symptoms related to metastatic PC, between 2013 and 2016, at the Department of Orthopedics, Sahlgrenska University Hospital, Gothenburg. The study protocol was approved by the ethics committee in Gothenburg, Sweden (# 936-12 and # 455-11). We excluded two patients that had other cancer diagnoses in addition to PC and one patient that was CTC-negative. Clinical information of the included 22 patients is shown in Table 1. The hormone naïve patients (n=5) were diagnosed with metastatic PC at the time of surgery and did not receive any hormonal therapy before surgery. The patient annotated as GnRH naïve in this study received bicalutamide three months before surgery, but responded to GnRH therapy initiated after surgery. One patient annotated as GnRH initiated in this study was diagnosed with metastatic PC and was treated with GnRH antagonists for one month before surgery, after which he responded well to GnRH agonists. The CRPC patients (n=15) relapsed with metastasis in the spine after GnRH therapy alone, or after second or third line therapy (Table 1).

### RNA preparation and cDNA synthesis from metastatic tissue

Metastatic tissue that was removed during surgery was immersed in RNA*later* (Ambion) and frozen at -80°C. Total RNA was prepared from 40-100 mg tissue (dependent on the content of bone) using the RNeasy Plus Universal Mini kit (Qiagen) according to the manufacturer's instructions. Briefly, the tissue was homogenized in the Qiazol Lysis Reagent in a Tissue Lyser II homogenizer at 25 Hz for5 min twice, and the resulting homogenate was treated with gDNA eliminator solution (Qiagen), extracted with chloroform. After centrifugation, the aqueous phase was mixed with 70% ethanol and directly loaded onto the RNeasy Mini spin column. After washing, the total RNA was eluted from the column in 30 μl RNase-free water, and its concentration and purity were measured in a NanoDrop (Thermo Scientific, Waltham, MA, USA). Forty nanograms of the RNA from metastatic tissue samples were converted to cDNA using the TATAA GrandScript cDNA Synthesis Kit (Cat. No. #A103a, TATAA Biocenter, Gothenburg, Sweden).

### CTC isolation and cDNA synthesis

The CTCs were isolated from blood samples and detected using the AdnaTest ProstateCancerSelect/Detect kit (Qiagen Hannover GmbH, Germany) as previously described [3]. Briefly, the patient blood samples were collected immediately before surgery in AdnaCollect tubes and refrigerated at 4°C. CTC isolation was performed within 24 hours by capturing them on EPCAM- and HER2 antibody-conjugated magnetic beads. The mRNAs from lysed CTCs were isolated using oligodT-conjugated magnetic beads and transcribed into cDNA.

### Design of the PC gene expression panel

As shown in Table 2, the gene expression panel, which is referred to as PC-panel, was composed of 46 genes, of which five were control genes, and 41 were PC-related genes. The genes were selected based on their role in PC progression, metastasis, steroid synthesis and signaling, stemness and neuroendocrine differentiation as reported in the literature. All the included genes were expected to be highly expressed in PC cells and negligible expression in the leukocytes (contaminating white blood cells in the CTC samples).

### Gene expression profiling

We pre-amplified 2 μl of cDNA samples from metastases and CTC samples (including beads from CTC collection) using the TATAA PreAmp Primer Mix and TATAA PreAmp GrandMaster^®^ Mix (Cat. No. #TA05, TATAA Biocenter) in a T100 BioRad thermocycler. We also pre-amplified non-template control and human gDNA (0.5 ng/μl, TATAA Biocenter) samples. The preamplified samples were centrifuged to pellet the magnetic beads, and a fraction of the supernatant was diluted 10X in a separate tube. We performed qPCR analyses of the diluted samples (45 assays) using the ValidPrime™ assay kit (TATAA Biocenter) with specific primers designed for this study as shown in Supplementary Table 1. This assay is now available as part of the GrandPerformance CTC Assay Panel at the TATAA Biocenter. The qPCR analysis was performed using the TATAA Probe GrandMaster^®^ Mix Low ROX (TATAA Biocenter) and GE 96.96 Dynamic Array™ Sample & Assay Loading Reagent Kit (P/N 85000802-R, Fluidigm). We also included preamplified no template control (preAmp NTC) and no template control (NTC) for the qPCR, which was performed on the BioMark system (Fluidigm) using the 96.96 Dynamic Array™ IFC (Integrated Fluidic Circuit). All the samples (including the NTCs and gDNA) were analyzed in duplicates. The assays we use were designed with ISO 17025 accredited methods (TATAA Biocenter) and validated in compliance with the MIQE guidelines [21], which is considered sufficient for research and most diagnostic usage.

### Preprocessing, normalization, and interpretation of gene expression data

The raw data (averaged Cq-values) was controlled and corrected for genomic DNA contamination using the GenEx software (MultiD Analyses AB) with implemented functions for the ValidPrime™ concept [22]. The averaged Cq values corrected with more than one cycle (Cq) were considered compromised due to large-scale genomic DNA contamination and removed from the analysis.

*EPCAM* expression was considered as a measure of epithelial cell (i.e. CTC) content. The expression of other genes was normalized to *EPCAM* expression to eliminate the contamination from normal bone tissue or white blood cells in metastatic tissue and CTC samples, respectively. When expression was not detected for some genes in the CTC samples, the results were not automatically interpreted as displaying low expression. Instead, we individually identified a cut-off level of CTC content (based on *EPCAM* expression) so that the gene expression levels could be reliably detected in CTC samples, and eliminate false low CTC expression values. If the CTC content was now high enough for the gene to be reliably detected, the low expression value was used instead of the absent signal. On the other hand, if the CTC content was lower than the cut-off detection level that was required to detect the expression of a particular gene, the absent signal was excluded from further analyses. Therefore, if a specific gene was not detected in a specific CTC sample, the Cq(*EPCAM*) value for that CTC sample was compared to the Cq(*EPCAM*) values in other CTC samples where the specific gene was detected. We interpreted the absent signal as a valid detection value if the Cq(*EPCAM*) value in the specific sample was at least 2Cq values lower (i.e. four times higher *EPCAM* expression) than the highest Cq(*EPCAM*) value at which the specific gene could be detected in all the CTC samples analyzed. In such a scenario, the expression of that specific gene was assigned a delta Cq value, which was one Cq value higher than the highest Cq value detected for that specific gene. If the Cq(*EPCAM*) value in the CTC sample was higher than the cut-off Cq(*EPCAM*) value, i.e., displaying a lower *EPCAM* expression, the absent signal was regarded as absent and excluded from further analyses.

### Statistics

Spearman Rank Correlation was used to compare the gene expression levels in CTC and metastasis samples from individual patients using the IBM SPSS Statistics 22.0.0.0 software. Since expression levels of the genes included in the panel may partly be dependent on each other within individual patients, the p-values were derived using the bootstrapping method as follows: Two patients were sampled randomly and the correlation between the CTC expression values from one patient and the metastasis expression values from the second patient were calculated. This was repeated 100000 times to achieve the empirical distribution function for the correlation between independent individuals. The p-value was then derived by comparing the observed correlations within patients to this distribution. The procedure was applied to the number of available complete pairs of matched samples for each patient. Hierarchical clustering was performed using normalized and mean centered data with Spearman Rank Correlation and Average linkage in a MultiExperiment Viewer (MeV, Dana-Farber Cancer Institute, US). All statistical tests were two-sided and p-values < 0.05 were considered statistically significant.

## SUPPLEMENTARY MATERIALS AND TABLES





## Abbreviations

CTC: Circulating tumor cells
CR: Castration resistant
HN: Hormone naïve
PC: Prostate cancer
Cq: Cycles of qPCR reaction
qPCR: quantitative real-time polymerase chain reaction
CRPC: Castration-resistant prostate cancer
GnRH: Gonadotropin-releasing hormone
AR: androgen receptor
RNA: ribonucleic acid
DNA: deoxyribonucleic acid
cDNA: Cyclic-DNA
