# Supplementary material for: Circulating tumor cells mirror bone metastatic phenotype in prostate cancer
Source: Oncotarget. 2018 Jun 29;9(50):29403–13. doi: 10.18632/oncotarget.25634 (PMC6047665; doi:10.18632/oncotarget.25634)
Supplement: Supplementary file 1 [file oncotarget-09-29403-s001.pdf]

Circulating tumor cells mirror bone metastatic phenotype in prostate cancer

SUPPLEMENTARY MATERIALS

Supplementary Table 1: Table of context sequences, assay names, efficiency and amplicon lengths for the selected TATAA Grand Performance assays.

See Supplementary File 1
